# Image-based assessment of aortoiliac aneurysm anatomical characteristics in patients from the global iliac branch study

**DOI:** 10.1007/s00423-024-03326-8

**Published:** 2024-04-23

**Authors:** Alina-Marilena Bresler, Annalise Panthofer, Yuki Kuramochi, Sydney L. Olson, Matthew Eagleton, Darren B. Schneider, Sean P. Lyden, William C. Blackwelder, Christian F. Uhl, Moritz S. Bischoff, Jon S. Matsumura, Dittmar Böckler

**Affiliations:** 1 Department of Vascular and Endovascular Surgery, Heidelberg University Hospital, Im Neuenheimer Feld 420, 69120 Heidelberg, Germany; 2 Department of Surgery, Division of Vascular Surgery, University of Wisconsin School of Medicine and Public Health, Madison, WI USA; 3 Vascular Surgery Department, Heart Vascular Thoracic Surgery Institute, Cleveland Clinic, Cleveland, OH USA; 4 Department of Surgery, Division of Vascular and Endovascular Surgery, Massachusetts General Hospital, Boston, MA USA; 5 Department of Surgery, Division of Vascular Surgery, Hospital of the University of Pennsylvania, Philadelphia, PA USA; 6 Department of Epidemiology and Public Health, University of Maryland School of Medicine, Baltimore, MD USA; 7 Department of Surgery, Division of Vascular Surgery, University of Colorado School of Medicine, Aurora, CO USA; 8 Department of Vascular Surgery, University Hospital RWTH Aachen, Aachen, Germany

**Keywords:** Iliac branch device (IBD), Iliac anatomy, Unilateral und bilateral iliac lesions

## Abstract

**Objective:**

Endovascular repair is the preferred treatment for aortoiliac aneurysm, with preservation of at least one internal iliac artery recommended. This study aimed to assess pre-endovascular repair anatomical characteristics of aortoiliac aneurysm in patients from the Global Iliac Branch Study (GIBS, NCT05607277) to enhance selection criteria for iliac branch devices (IBD) and improve long-term outcomes.

**Methods:**

Pre-treatment CT scans of 297 GIBS patients undergoing endovascular aneurysm repair were analyzed. Measurements included total iliac artery length, common iliac artery length, tortuosity index, common iliac artery splay angle, internal iliac artery stenosis, calcification score, and diameters in the device's landing zone. Statistical tests assessed differences in anatomical measurements and IBD-mediated internal iliac artery preservation.

**Results:**

Left total iliac artery length was shorter than right (6.7 mm, *P* = .0019); right common iliac artery less tortuous (*P* = .0145). Males exhibited greater tortuosity in the left total iliac artery (*P* = .0475) and larger diameter in left internal iliac artery's landing zone (*P* = .0453). Preservation was more common on right (158 unilateral, 34 bilateral) than left (105 unilateral, 34 bilateral). There were 192 right-sided and 139 left-sided IBDs, with 318 IBDs in males and 13 in females.

**Conclusion:**

This study provides comprehensive pre-treatment iliac anatomy analysis in patients undergoing endovascular repair with IBDs, highlighting differences between sides and sexes. These findings could refine patient selection for IBD placement, potentially enhancing outcomes in aortoiliac aneurysm treatment. However, the limited number of females in the study underscores the need for further research to generalize findings across genders.

**Supplementary information:**

The online version contains supplementary material available at 10.1007/s00423-024-03326-8.

## Introduction

In up to 40% of cases of patients with abdominal aortic aneurysm (AAA), there is a coexistence of common iliac artery aneurysm (CIAA) [1]. In recent years, significant advancements have been made in endovascular techniques, with the preservation of the internal iliac artery (IIA) patency emerging as a crucial step. The conventional practice of routinely embolizing the IIA in most cases has been replaced by the utilization of side branch techniques, which prioritize the maintenance of IIA openness. The preservation of at least one internal iliac artery is important to reduce the risk of complications associated with perfusion of pelvic organs and muscles [2]. The European Society of Vascular Surgery recommends that endovascular repair should be regarded as the primary treatment option for patients with iliac artery aneurysm [3]. Iliac branch devices (IBD) have been developed to facilitate endovascular repair while maintaining antegrade flow into the internal iliac artery [4]. The successful treatment of patients with IBD relies heavily on the anatomical fit between the patients and the implanted device. As a result, improving the selection criteria based on anatomical suitability has the potential to significantly improve long-term outcomes. There are several well- known risk factors that can contribute to the failure of implantation, including the presence of a narrowed lumen in the common iliac artery, severe kinking of the external iliac artery, a stenotic internal iliac ostium, and wide angle (> 50°) in the branching of the internal iliac artery [5]. However, the current literature does not provide sufficient description of these characteristics.

Utilizing a multi-center multi-country collaborative data set, the objective of this project was to capture pre-endovascular repair anatomical characteristics of aortoiliac aneurysm in patients from the Global Iliac Branch Study (GIBS, NCT05607277), in order to identify unique features relevant to stent-graft planning and application.

## Methods

### Study design

This is an international, multicenter, explorative, retrospective study of 297 patients with coexisting bilateral or unilateral common iliac artery aneurysms. Data was collected from the GORE EXCLUDER prospective, multicenter 12–04 trial (NCT01883999) (W.L. Gore & Associates, Flagstaff, Ariz), the Cleveland Clinic, North America and Heidelberg University Hospital, Germany. Inclusions criteria were patients undergoing evaluation for EVAR with a main trunk AAA device along with an iliac branch device on at least one side and available pre-treatment CTA imaging. All patients who underwent endovascular or open repair for aortoiliac aneurysm rupture, pseudoaneurysm, solitary CIA aneurysm, or mycotic aneurysm were excluded from this study.

### Computer tomography measurements

**Fig. 1:**
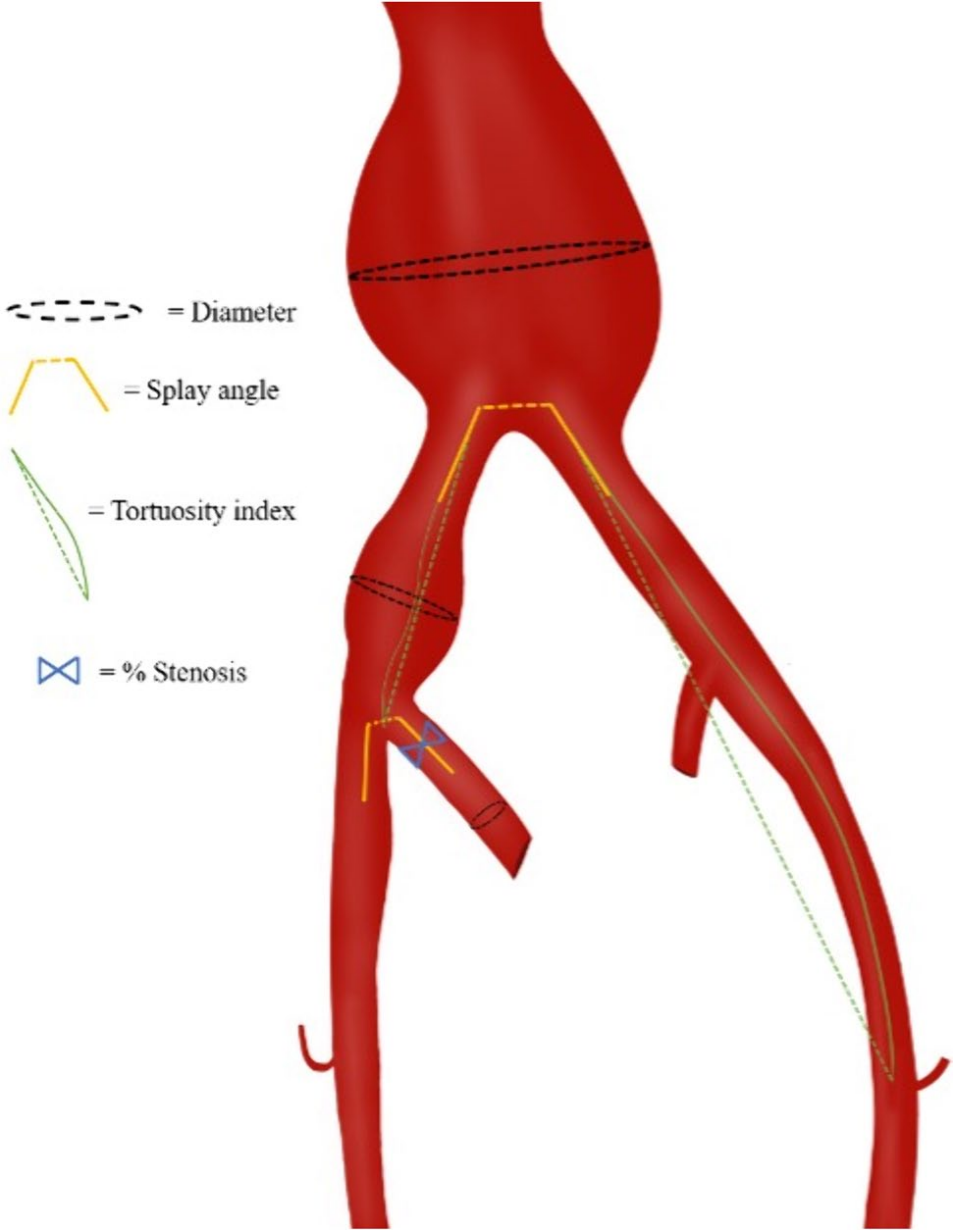
Summary of pre-treatment measurements

The measurements were performed at the University of Wisconsin AortaCore Aortic Imaging Lab under an Institutional Review Board-approved protocol using Aquarius iNtuition (version 4.4.12, TeraRecon). Trained image processors utilized automated centerlines, which were manually adjusted as necessary, for all images. Measurements were then carried out by image analysts who underwent regular proficiency testing. To ensure consistency and accuracy, the measurements were carefully assessed and validated for interobserver reliability. A Summary of location of pre-treatment measurements are included in Fig. 1.

### Definitions and outcome measures

**Fig. 2:**
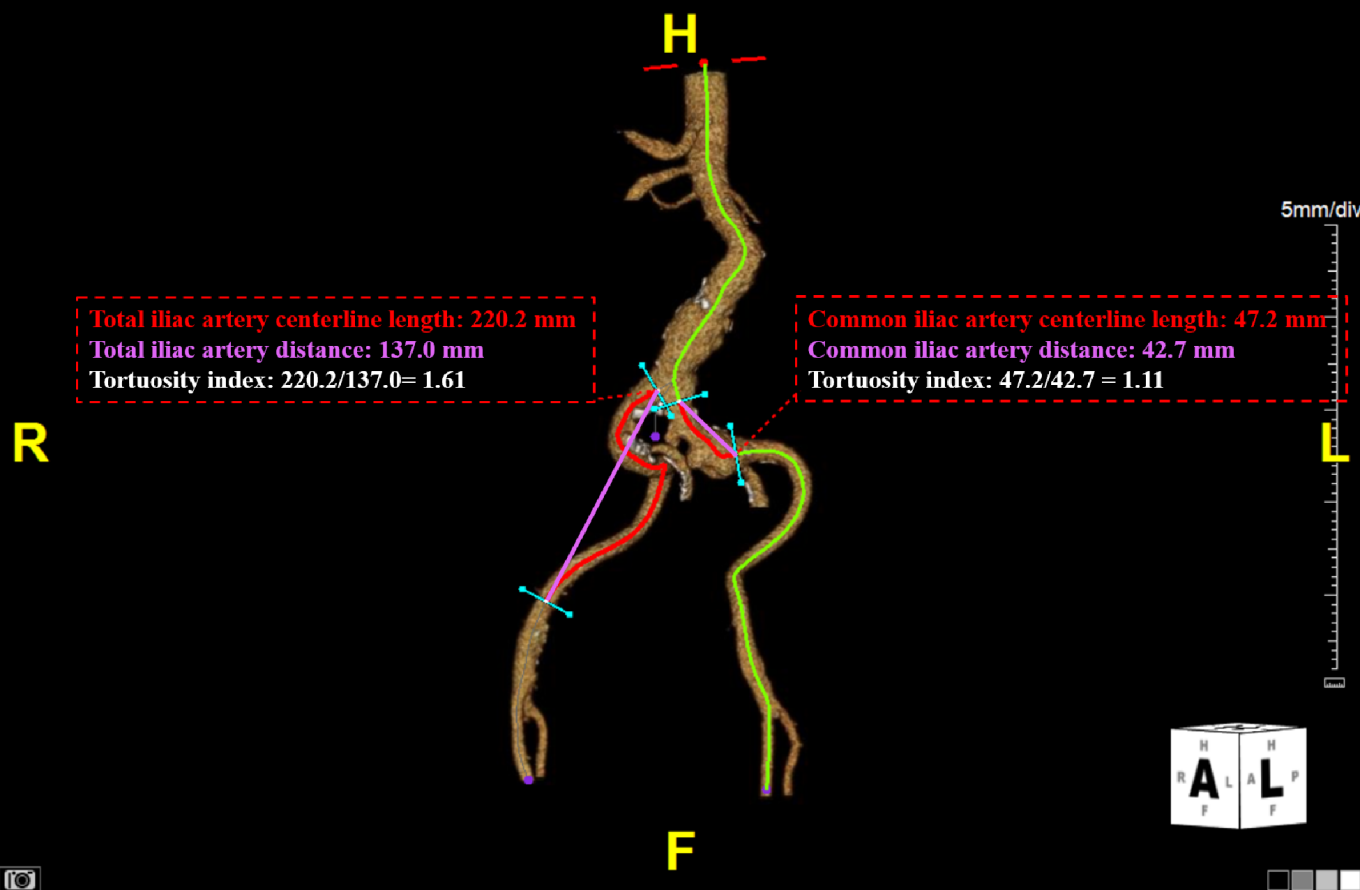
Measurements performed bilaterally in all patients included total iliac artery length, total iliac artery distance, total iliac artery tortuosity index, common iliac artery length, common iliac artery distance, and common iliac artery tortuosity index

Bilateral measurements performed in all patients’ right and left iliac arteries included length of the total iliac artery, total iliac artery tortuosity index, length of the common iliac artery, and common iliac artery tortuosity index (Fig. 2). Total iliac artery tortuosity index was calculated by centerline distance between the beginning of the common iliac artery to the inferior epigastric artery origin, divided by straight distance between these two points. Common iliac artery tortuosity index was calculated by centerline distance between the start of the common iliac artery to the iliac bifurcation, divided by straight distance between these two points. A value closer to 1.0 indicates less tortuosity, whereas higher values indicate more tortuous iliac anatomy.

**Fig. 3:**
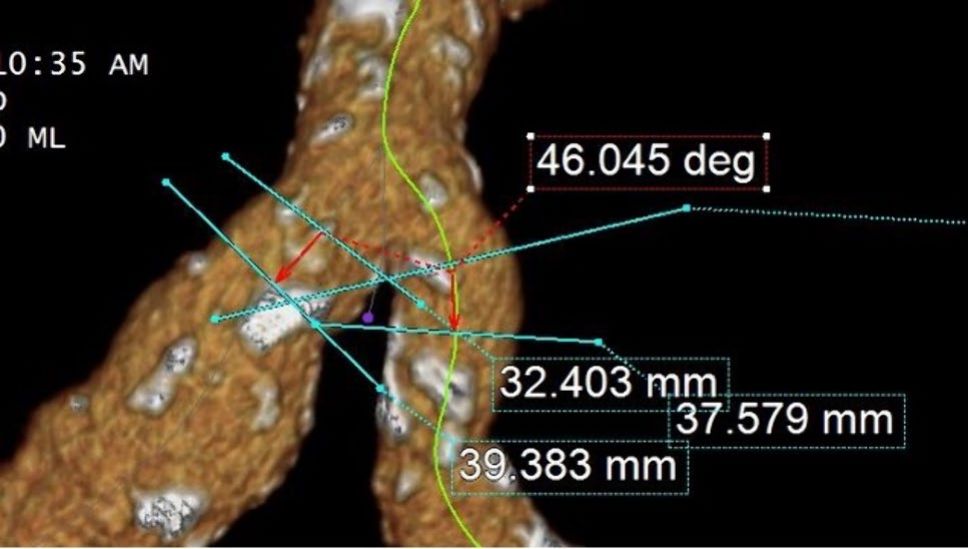
Aortic Bifurcation Splay Angle

Ipsilateral measurements performed only on patient’s treatment-side included common iliac artery splay angle, approximate internal iliac artery stenosis, calcification score, common iliac aneurysm maximum transverse diameter, and internal iliac diameter in approximate distal landing zone of device. Common iliac artery splay angle was determined by measuring the angle formed by extending a 10 mm distance ray from first slice of common distinct blood flow without going over. To measure the angle, points were marked at the distal end of one iliac artery, the proximal end of the same iliac artery, and the distal end of the other iliac artery. Then, by dragging the proximal point, a dashed connecting line was created, splitting the line into two sections. Iliac splay angle was measured using the same technique at the level of the iliac bifurcation (Fig. 3). The maximum transverse diameter of the abdominal aortic aneurysm and the common iliac artery aneurysm was measured outer-wall-to-outer-wall in the orthogonal view, perpendicular to the centerline. The diameter of the common iliac artery was measured outer-wall-to-outer-wall, 15 mm before the iliac bifurcation. This measurement serves as a substitute for the common iliac artery diameter, as stated in the instructions for use of medical devices.

### Study objectives

The main objective was to evaluate anatomical disparities between the left and right iliac sides. Secondary objectives included comparing iliac anatomy variations between males and females, investigating differences in iliac centerline lengths and indices on the same side versus the opposite side.

### Statistical analysis

The results were reported by hazard ratios (HRs) with corresponding 95% confidence intervals (CIs). To identify dissimilarities in the preservation of the internal iliac artery on the right versus left sides due to IBD, the chi-square test was employed. Unpaired t-tests were conducted to determine differences in the average measurements of iliac anatomy between the right and left sides. In addition, unpaired t-tests were used to detect differences in these measurements between males and females. To compare ipsilateral versus contralateral iliac centerline lengths and indices, t-tests with 95% CIs were performed. Statistical analyses were performed using GraphPad Prism version 9.5.1 (733).

## Results

This study enrolled 297 patients between 2004 and 2020, whom had pre-treatment CT imaging scans available and of adequate quality for performing accurate measurements. The mean age of all subjects was 70.6 years. There were 286 male and 11 female subjects included.

A summary of anatomic comparisons between subjects treated for left versus right anatomy are presented in Table 1. The median size of the common iliac artery aneurysm was 38.1 mm on the right side and 37.7 mm on the left side. The left total iliac artery was found to be shorter by 6.7 mm (*P* = 0.0019, unpaired t-test) compared to the right, whereas the right common iliac artery was observed to be less tortuous than the left (1.10 vs. 1.13, *P* = 0.0145). No disparities were identified between left and right total iliac artery tortuosity index. In relation to the common iliac artery splay angle, there was a difference of 2.1° with the left common iliac artery being smaller than the right.

**Table 1:** Right versus Left Iliac Anatomy

Bilateral measurements performed in 297 patients				
	Right	Left	Difference [95% CI]	*P*-value
	(*n* = 297)	(*n* = 297)		
Total iliac artery centerline length (mm)	183.2	176.5	−6.7 [ −10.9 to −2.5]	.0019**
Total iliac artery tortuosity index	1.46	1.46	0 [ −.04 to .04]	.9134
Common iliac artery centerline length (mm)	60.6	60.9	.3 [ −3.4 to 3.8]	.9032
Common iliac artery tortuosity index	1.10	1.13	.03 [.01 to .05]	.0145*
Ipsilateral measurements performed in 331 iliac arteries				
	Right	Left	Difference [95% CI]	*P*-value
	(*n* = 192)	(*n* = 139)		
Common iliac artery splay angle (deg)	51.4	53.5	2.1 [ −3.0 to 7.3]	.4151
Approximate % stenosis of internal iliac	11.0	11.7	.7 [ −3.2 to 4.5]	.7273
Calcification score	2.2	2.1	−.1 [ −.2 to .2]	.7914
Common iliac artery aneurysm max. diameter (mm)	38.1	37.7	−.4 [ −2.9 to 2.3]	.7976
Internal iliac diameter in distal landing zone (mm)	9.8	9.9	.1 [ −.4 to .6]	.6831

Gender-specific dimensions of the iliac anatomic segments are presented in Table 2. Among males, the median size of the right common iliac artery aneurysm was 38.1 mm, while in females it was 35.5 mm. For the left common iliac artery aneurysm, the median size was 37.8 mm in males and 36.8 mm in females. Specifically, the analysis demonstrated that the tortuosity index of the left total iliac artery was significantly higher in males than in females (1.47 vs. 1.32, *P* = 0.0475). Moreover, the diameter of the left internal iliac artery in the distal landing zone was found to be significantly larger in males than in females (10.0 mm vs. 8.0 mm, *P* = 0.0453).

**Table 2:** Male versus Female Iliac Anatomy

Bilateral measurements performed in 297 patients				
	Male	Female	Difference [95% CI]	*P*-value
	(*n* = 286)	(*n* = 11)		
Right total iliac artery centerline length (mm)	183.6	171.2	−12.4 [ −27.2 to 2.8]	.1098
Left total iliac artery centerline length (mm)	177.0	163.5	−13.5 [ −29.7 to 2.7]	.1031
Right total iliac artery tortuosity index	1.46	1.39	−.07 [ −.21 to .07]	.3121
Left total iliac artery tortuosity index	1.47	1.32	−.15 [ −.30 to −.002]	.0475*
Right common iliac artery centerline length (mm)	60.9	54.2	−6.7 [ −20.4 to 7.0]	.3381
Left common iliac artery centerline length (mm)	61.0	58.1	−2.9 [ −16.3 to 10.5]	.6712
Right common iliac artery tortuosity index	1.10	1.08	−.02 [ −.08 to .05]	.5951
Left common iliac artery tortuosity index	1.13	1.11	−.02 [ −.13 to .09]	.7063
Ipsilateral measurements performed in 331 iliac arteries				
	Male	Female	Difference [95% CI]	*P*-value
	(*n* = 318)	(*n* = 13)		
Right common iliac artery splay angle (deg)	51.5	46.3	−5.2 [ −24.3 to 13.9]	.5901
Left common iliac artery splay angle (deg)	53.3	57.0	3.7 [ −14.9 to 22.3]	.6923
Combined common iliac artery splay angle (deg)	52.3	52.1	−.2 [ −13.4 to 13.0]	.9786
Right approximate % stenosis of internal iliac	10.9	16.7	5.8 [ −8.6 to 20.2]	.4271
Left approximate % stenosis of internal iliac	11.9	8.6	−3.3 [ −16.9 to 10.3]	.6299
Combined approximate % stenosis of internal iliac	11.3	12.3	1.0 [ −8.8 to 10.8]	.8383
Right calcification score	2.2	2.2	0 [ −.7 to .7]	.9764
Left calcification score	2.1	2.4	.3 [ −0.4 to 1.0]	.3968
Combined calcification score	2.1	2.3	.2 [ −.3 to .7]	.5099
Right common iliac artery aneurysm max. diameter (mm)	38.1	35.5	−2.6 [ −12.4 to 7.1]	.5903
Left common iliac artery aneurysm max. diameter (mm)	37.8	36.8	−1.0 [ −10.0 to 8.2]	.8423
Combined common iliac artery aneurysm max. diameter (mm)	38.0	36.2	−1.8 [ −8.4 to 4.8]	.5985
Right internal iliac diameter in distal landing zone (mm)	9.8	10.0	.2 [ −1.4 to 1.7]	.8651
Left internal iliac diameter in distal landing zone (mm)	10.0	8.0	−2.0 [ −3.9 to −.04]	.0453*
Combined internal iliac diameter in distal landing zone (mm)	9.9	8.9	−1.0 [ −2.2 to .2]	.1135

Table 3 summarizes differences seen in ipsilateral vs contralateral iliac centerline lengths and indices. The ipsilateral total iliac artery length was longer for the ipsilateral compared to the contralateral group (178 mm vs. 175,2 mm, *P* = 0,201). However, the common iliac artery length was found to be shorter in the ipsilateral group compared to the contralateral group (56,78 mm vs 57,73 mm). The common iliac artery tortuosity was not significant for the ipsilateral group compared to the contralateral group. Common iliac artery tortuosity index was similar between the two groups. In contrast, the contralateral common iliac artery tortuosity index was higher compared to the ipsilateral group (1,060 vs 1,070, *P* = 0,049).

**Table 3:** Ipsilateral vs contralateral centerline lengths and indice

	Ipsilateral iliac (*n* = 263)	Contralateral iliac (*n* = 263)	Difference [95%]	*P*-value
Total iliac artery centerline length (mm)	178.0	175.2	2.953 [.4669 to 5.440]	.0201*
Total iliac artery tortuosity index	1.427	1.411	.01271 [.006939 to .03236]	.2039
Common iliac artery centerline length (mm)	56.78	57.73	1.962 [.2585 to 4.182]	.0831
Common iliac artery tortuosity index	1.060	1.070	.02178 [.04348 to −8.997e −005]	.0491*

In this study, 154 out of 286 males were evaluated for unilateral right-sided IBDs, while 32 males were evaluated for bilateral IBDs, and 100 males were evaluated for unilateral left-sided IBDs. Among the 11 females, 4 were evaluated for unilateral right-sided IBDs, 2 for bilateral IBDs, and 5 for unilateral left-sided IBDs. A total of 331 Iliac Branch Devices (IBDs) were planned in this study, with 192 being right sided and 139 being left sided. Among these, 158 were unilateral right-sided IBDs, 34 were bilateral IBDs and 105 were unilateral left-sided IBDs (Supplemental Fig. 1). IBD-mediated preservation of the internal iliac artery was more common on the right than the left (*P* < 0.00001, chi-square test).

By individual iliac branch system, there were 192 right-sided IBDs and 139 left-sided IBDs planned. There were 286 males, 154 were evaluated for unilateral right-sided IBD, 100 were for unilateral left-sided IBD, and 32 were for bilaterally IBDs (318 IBD, 186 right-sided IBD, 132 left-sided IBD). For 11 females were 13 IBDs planned (6 right-sided IBD, 7 left-sided IBD).

## Discussion

Our study showed that, the dimensions of the left iliac artery are typically smaller than those of the right iliac artery. Furthermore, upon conducting a gender-specific analysis, we found that this size disparity persists, with the left iliac artery consistently exhibiting smaller dimensions compared to its right counterpart. In a study encompassing a cohort of healthy individuals from the Asian population, utilizing CTA, it was determined that the diameter of the CIA exhibited measurements of 11.436 ± 1.512 mm among males and 9.793 ± 1.464 mm among females. Notably, across all assessed mean diameters and lengths, noteworthy distinctions were evident between males and females, except for both CIA lengths [6]. The success of endovascular treatment depends on appreciation of the anatomy of the target vascular segment. Femoral and iliac artery entry points are crucial for the placement of aortic and iliac devices. The characteristics of the common femoral and iliac arteries are important in allowing vascular access. Calcifications and constricted, twisting vessels could result in the occlusion of limbs, necessitating additional intervention [7]. Those findings are corroborated by the study conducted by Fenelli et al., wherein they demonstrated that a preoperative pelvic tortuosity index > 1.4 an independent predictor of overall complications and the likelihood of subsequent IBD interventions [8]. An earlier study by Ziegler et al. involving 51 attempted cases revealed an increased likelihood of encountering implantation challenges and a higher risk of failure due to the factors mentioned previously [5]. However, due to our diminished interobserver reliability regarding the double iliac sign, we are unable to assess the latter of these findings. It is noteworthy that our study, featuring a sample size six times larger, did not establish the common iliac artery tortuosity index as a predictive factor for overall adverse iliac events in the context of IBDs. A case control design has been used to investigate CIAA characteristics which can be easily detected in the pre-operative CTA and which may predispose to post-operative complications. The present anatomic analysis showed an average of right CIA length of 60,9 mm in male and 54,2 mm in female which was shorter than the left CIA length in male and female patients (61 mm and 58,1 mm). Our findings align also with a case report conducted by Fereydooni et. al. which showed that the rights CIA length was shorter that the left CIA length in male (87,8 mm vs 100 mm) [9]. Similarly, a study of 3,692 patients who underwent computed tomography as part of a general health checkup from 2015–2019 in a single tertiary center from Kim et. al., it was found that the average length of the right CIA was shorter in females (47.9 mm) and males (49 mm) compared to the length of the left CIA (52.7 mm in females and 53.9 mm in males) [6]. In comparison to previously reported lengths in Asian patients, our anatomic analysis exhibited a greater length, while still being shorter than what is described in the published literature for CIA aneurysms [10–13].

The presence of Iliac tortuosity has been associated with a heightened potential for arterial dissection, rupture, limb bending, or blockage, as indicated by previous studies [14, 15]. The preoperative CTA assessment included an individual evaluation of the tortuosity of CIA and stenosis of native CIA. Our patients showed bigger common iliac arteries (38,1 mm right and 37,7 mm left) and less tortuous (1.10 right and 1.13 left) compared to Wyss et al. which reported a common iliac artery tortuosity index of 1.2 ± 0,2 and a common iliac artery diameter of 16 mm [16]. D’Oria et al. reported a comparable size for the diameter of the common iliac artery in our patients, measuring approximately 37 ± 10 mm [17]. Lee et.al. reported a common iliac artery tortuosity index of 1.43. Thus, our patients have less tortuous common iliac arteries than those of Lee et.al [18]. The present study also revealed the differences that exist between the common iliac artery tortuosity index in females and males. Our study found common iliac artery tortuosity in males was more tortuous then in females (Table 2).

### Study limitations

The study was limited by retrospective data collection and relied on clinical trial registry data, which may be susceptible to errors in registration and potential biases in patient selection and referral patterns. The small sample size of females and measurements within the female subgroups prevented further statistical evaluation of disparities between genders. However, the inclusion of multiple centers strengthens the overall generalizability of the study, but it also introduces heterogeneity due to the lack of documented turn-down rates and various follow-up protocols. Finally, although all participating centers have experience in complex endovascular aortic procedures, there were significant differences in patient volume across the centers.

## Conclusions

This project provides a robust overview of pre-treatment iliac anatomy in patients undergoing endovascular repair with iliac branch devices for the treatment of aortoiliac aneurysm. There remains a notable gender disparity in the literature concerning aortoiliac aneurysms, with insufficient attention directed towards reporting and analyzing sex-specific and gender-related differences in research. Discrepancies in length, diameter, and tortuosity should be considered in bilateral IBD planning, especially in females due to gender differences. More data on aortoiliac aneurysms in women are needed. These findings may be useful for clinicians and researchers in developing strategies for improving the safety and efficacy of endovascular repair procedures for this patient population.

### Supplementary information

Below is the link to the electronic supplementary material.

Supplementary file1 (DOCX 139 KB)

## References

[1] Richards T, Dharmadasa A, Davies R, Murphy M, Perera R, Walton J (2009). Natural history of the common iliac artery in the presence of an abdominal aortic aneurysm. J Vasc Surg.

[2] Cao Z, Zhu R, Ghaffarian A, Wu W, Weng C, Chen X (2022). A systematic review and meta-analysis of the clinical effectiveness and safety of unilateral versus bilateral iliac branch devices for aortoiliac and iliac artery aneurysms. J Vasc Surg.

[3] Wanhainen A, Verzini F, Van Herzeele I, Allaire E, Bown M, Cohnert T (2019). Editor’s choice - European Society for Vascular Surgery (ESVS) 2019 clinical practice guidelines on the management of abdominal aorto-iliac artery aneurysms. Eur J Vasc Endovasc Surg Off J Eur Soc Vasc Surg.

[4] Chaikof EL, Dalman RL, Eskandari MK, Jackson BM, Lee WA, Mansour MA (2018). The Society for Vascular Surgery practice guidelines on the care of patients with an abdominal aortic aneurysm. J Vasc Surg.

[5] Ziegler P, Avgerinos ED, Umscheid T, Perdikides T, Erz K, Stelter WJ (2007). Branched iliac bifurcation: 6 years experience with endovascular preservation of internal iliac artery flow. J Vasc Surg.

[6] Kim H, Kwon T-W, Choi E, Jeong S, Kim H-K, Han Y (2022). Aortoiliac diameter and length in a healthy cohort. PLoS ONE.

[7] Woody JD, Makaroun MS (2004). Endovascular graft limb occlusion. Semin Vasc Surg.

[8] Fenelli C, Gargiulo M, Prendes CF, Faggioli G, Stavroulakis K, Gallitto E (2022). Effect of iliac tortuosity on outcomes after iliac branch procedures. J Vasc Surg.

[9] Fereydooni A, Deyholos C, Botta R, Nezami N, Dardik A, Nassiri N (2019). Bifurcated unibody aortic endografts can overcome unfavorable aortoiliac anatomy for deployment of bilateral iliac branch endoprostheses. J Vasc Surg cases Innov Tech.

[10] Gray D, Shahverdyan R, Jakobs C, Brunkwall J, Gawenda M (2015). Endovascular aneurysm repair of aortoiliac aneurysms with an iliac side-branched stent graft: studying the morphological applicability of the Cook device. Eur J Vasc Endovasc Surg Off J Eur Soc Vasc Surg.

[11] Itoga NK, Fujimura N, Hayashi K, Obara H, Shimizu H, Lee JT (2017). Outcomes of Endovascular Repair of Aortoiliac Aneurysms and Analyses of Anatomic Suitability for Internal Iliac Artery Preserving Devices in Japanese Patients. Circ J.

[12] Hwang D, Yun W-S, Kim H-K, Huh S (2023). Off-label use of an iliac branch device and a reversed iliac limb for a patient with a unilateral common iliac artery aneurysm and a narrow distal aorta: a case report. Medicine.

[13] Wang L, Shu C, Li Q, Li M, He H, Li X (2021). Application of a Novel Common-Iliac-Artery Skirt Technology (CST) in Treating Challenge Aorto-Iliac or Isolated Iliac Artery Aneurysms. Front Cardiovasc Med.

[14] Carroccio A, Faries PL, Morrissey NJ, Teodorescu V, Burks JA, Gravereaux EC (2002). Predicting iliac limb occlusions after bifurcated aortic stent grafting: anatomic and device-related causes. J Vasc Surg.

[15] Tillich M, Bell RE, Paik DS, Fleischmann D, Sofilos MC, Logan LJ (2001). Iliac arterial injuries after endovascular repair of abdominal aortic aneurysms: correlation with iliac curvature and diameter. Radiology.

[16] Wyss TR, Dick F, Brown LC, Greenhalgh RM (2011). The influence of thrombus, calcification, angulation, and tortuosity of attachment sites on the time to the first graft-related complication after endovascular aneurysm repair. J Vasc Surg.

[17] D’Oria M, Tenorio ER, Oderich GS, DeMartino RR, Kalra M, Shuja F (2022). Outcomes of unilateral versus bilateral use of the iliac branch endoprosthesis for elective endovascular treatment of aorto-iliac aneurysms. Cardiovasc Intervent Radiol.

[18] Lee H, Choi J, Han Y, Cho Y-P, Kwon T-W (2013). Tortuosity index and angulation of the common iliac artery in abdominal aortic aneurysm patients treated with the endovascular technique to provide adequate access route. Korean J Vasc Endovasc Surg.

