# Supplementary figures and images for: Image-based assessment of aortoiliac aneurysm anatomical characteristics in patients from the global iliac branch study
Source: Langenbecks Arch Surg. 2024 Apr 23;409(1):135. doi: 10.1007/s00423-024-03326-8 (PMC11035386; doi:10.1007/s00423-024-03326-8)

**
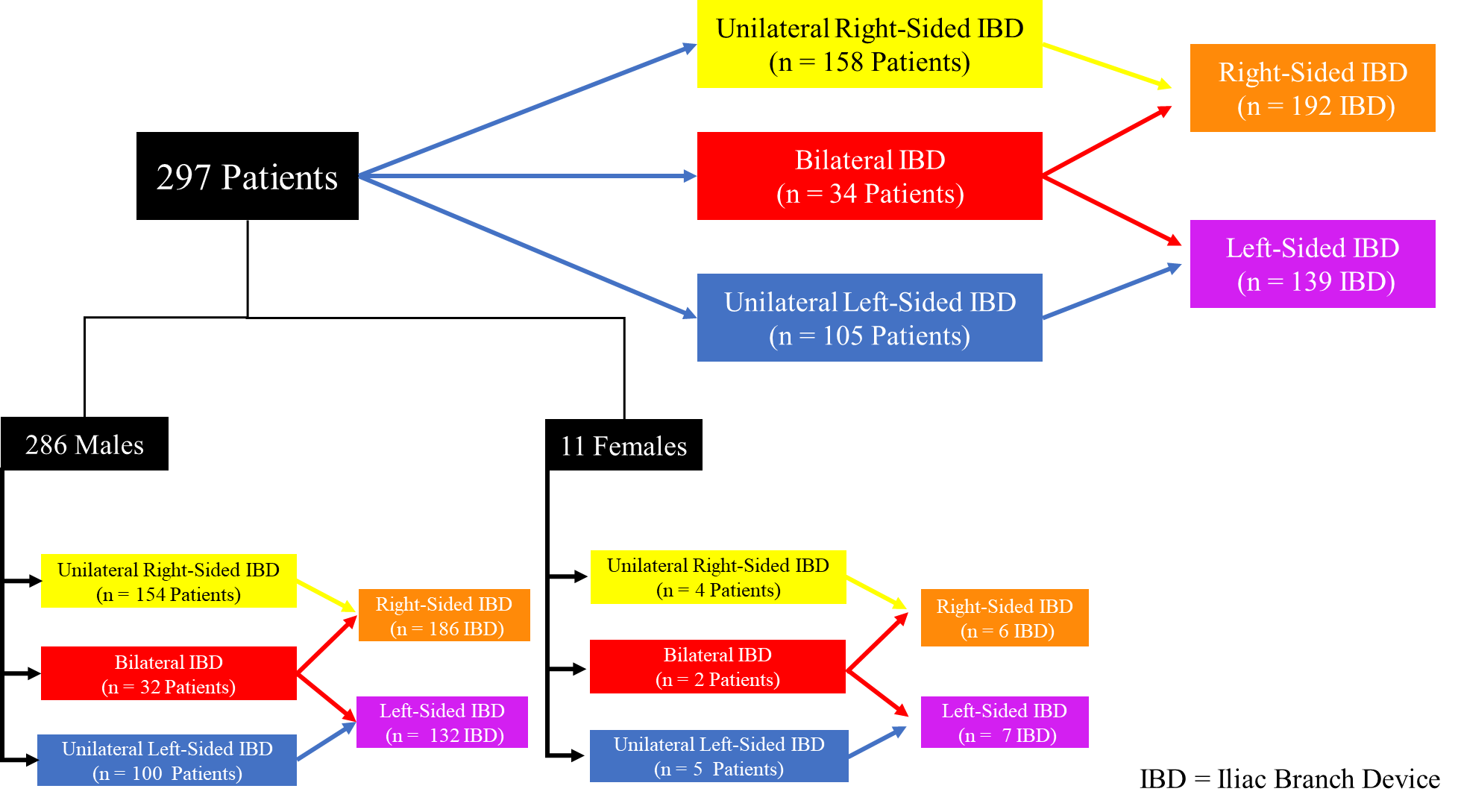
**

**Supplemental Figure 1. Number of Iliac Branch Devices**

Supplement: Supplementary file 1 — Supplementary file1 (DOCX 139 KB) [file 423_2024_3326_MOESM1_ESM.docx]
